# 6,7-Dichloro-3-(2,4-dichloro­benz­yl)­quinoxalin-2(1*H*)-one

**DOI:** 10.1107/S160053681203098X

**Published:** 2012-07-18

**Authors:** Jinpeng Zhang, Yinan Wang, Qian Wang, Lichun Xu

**Affiliations:** a Department of Public Health, Xuzhou Medical College, Xuzhou 221000, People’s Republic of China.; b Out Patient Department, Xuzhou Airforce College, Xuzhou 221000, People’s Republic of China.

## Abstract

In the title compound, C_15_H_8_Cl_4_N_2_O, the quinoxaline ring system is almost planar, with a dihedral angle between the benzene and pyrazine rings of 3.1 (2)°. The 2,4-dichloro­phenyl ring is approximately perpendicular to the pyrazine ring, with a dihedral angle of 86.47 (13)° between them. The crystal packing features inter­molecular N—H⋯O hydrogen bonds and π–π stacking inter­actions, with centroid–centroid distances in the range 3.699 (3)–4.054 (3) Å.

## Related literature

For the bioactivity of quinoxalin-2(1*H*)-one derivatives, see: Mensah-Osman *et al.* (2002 ▶); Perez *et al.* (2002 ▶); Quint *et al.* (2002 ▶); Seitz *et al.* (2002 ▶).
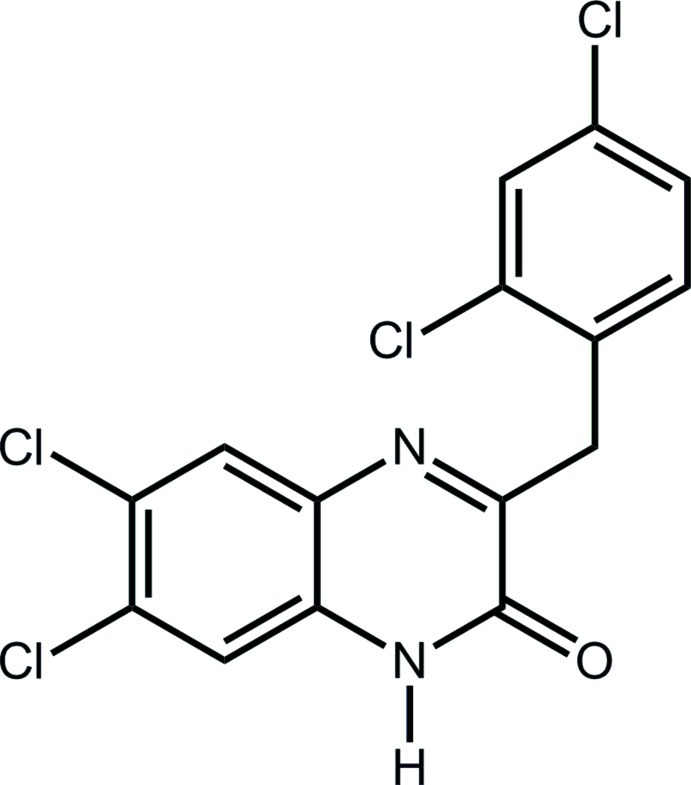


## Experimental

### 

#### Crystal data

C_15_H_8_Cl_4_N_2_O
*M* *_r_* = 374.03
Triclinic, 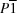

*a* = 7.7150 (7) Å
*b* = 8.2058 (8) Å
*c* = 11.9722 (12) Å
α = 83.771 (1)°
β = 84.362 (1)°
γ = 84.298 (2)°
*V* = 746.79 (12) Å^3^
*Z* = 2
Mo *K*α radiation
μ = 0.79 mm^−1^
*T* = 298 K
0.16 × 0.09 × 0.05 mm

#### Data collection

Bruker SMART CCD area-detector diffractometer
Absorption correction: multi-scan (*SADABS*; Sheldrick, 1996 ▶) *T*_min_ = 0.884, *T*_max_ = 0.961
3811 measured reflections
2590 independent reflections
1364 reflections with *I* > 2σ(*I*)
*R*_int_ = 0.036

#### Refinement

*R*[*F* ^2^ > 2σ(*F* ^2^)] = 0.055
*wR*(*F* ^2^) = 0.103
*S* = 1.01
2590 reflections
199 parameters
H-atom parameters constrained
Δρ_max_ = 0.27 e Å^−3^
Δρ_min_ = −0.26 e Å^−3^

### 

Data collection: *SMART* (Bruker, 1998 ▶); cell refinement: *SAINT* (Bruker, 1999 ▶); data reduction: *SAINT*; program(s) used to solve structure: *SHELXS97* (Sheldrick, 2008 ▶); program(s) used to refine structure: *SHELXL97* (Sheldrick, 2008 ▶); molecular graphics: *SHELXTL* (Sheldrick, 2008 ▶); software used to prepare material for publication: *SHELXTL*.

## Supplementary Material

Crystal structure: contains datablock(s) I, global. DOI: 10.1107/S160053681203098X/sj5253sup1.cif

Structure factors: contains datablock(s) I. DOI: 10.1107/S160053681203098X/sj5253Isup2.hkl

Supplementary material file. DOI: 10.1107/S160053681203098X/sj5253Isup3.cml

Additional supplementary materials:  crystallographic information; 3D view; checkCIF report

## Figures and Tables

**Table 1:** Hydrogen-bond geometry (Å, °)

*D*—H⋯*A*	*D*—H	H⋯*A*	*D*⋯*A*	*D*—H⋯*A*
N1—H1⋯O1^i^	0.86	1.93	2.789 (4)	173

Symmetry code: (i) 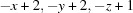.

**Fig. 1.**
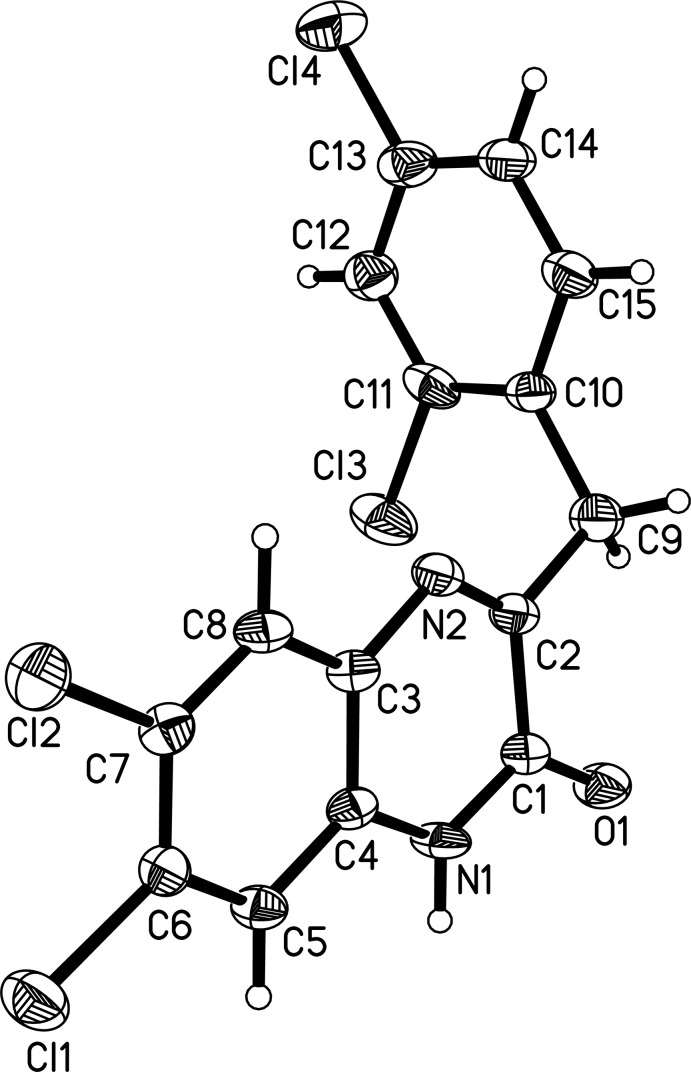
The structure of (I), showing 30% probability displacement ellipsoids and the atom-numbering scheme.

**Fig. 2.**
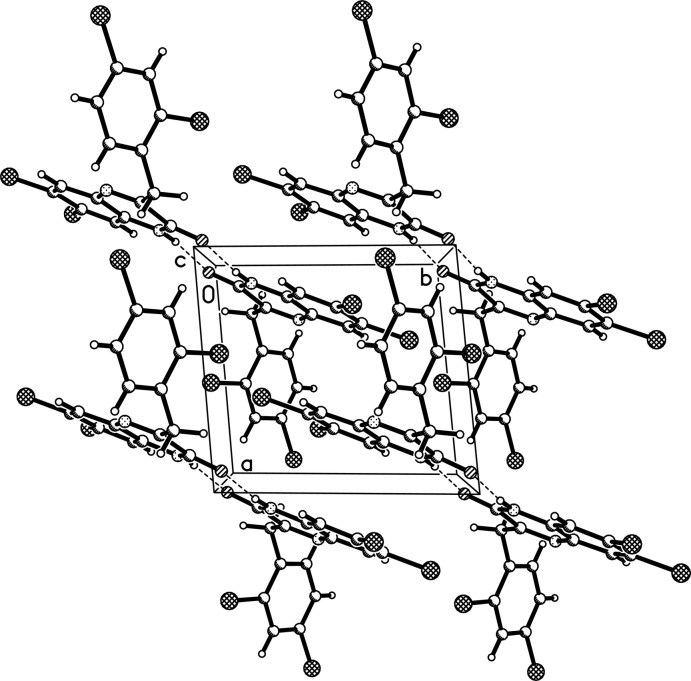
Crystal packing of (I), with hydrogen bonds drawn as dashed lines.

## supplementary crystallographic information

### Comment

Quinoxalin-2(1*H*)-one derivatives have attracted much attention in the pharmaceutical field due to their diverse bioactivities. These include use as a glutamate blocker (Perez *et al.* 2002), in the treatment of sensorineural smell disorders (Quint *et al.* 2002) and as a DNA topoisomerase (Topo) II beta-inhibitor (Mensah-Osman *et al.* 2002). They also exhibit antimycobacterial activity (Seitz *et al.* 2002). These reports inspired us to study the relationship between their structures and activities. During the synthesis of some quinoxalin derivatives, the title compound, (I) was isolated and its structure was confirmed by X-ray diffraction. Herein we report this structure.

In the molecular structure (Fig. 1), the quinoxaline ring system is nearly planar with a dihedral angle between the phenyl and pyrazine rings of 3.12(0.22) ° and rms deviations of 0.0135 Å and 0.0210 Å, respectively. The largest deviations from the planes of the two rings are 0.020 (3) Å for C3 and 0.031 (3) Å for C1. The 2,4-dichlorophenyl and pyrazine rings are approximately orthogonal with a dihedral angle of 86.47 (13) ° between them.

The crystal packing is stabilized by intermolecular N—H···O hydrogen bonds that form inversion dimers. In addition π–π stacking interactions are also found involving the C3–C8 and C10–C15 phenyl rings (Fig. 2). The centroid-to-centroid distances, plane-plane distances and displacement distances are as follows: 4.054 (3), 3.404 (2) and 2.201 Å (C3–C8 to C3–C8; symmetry code: 1-*X*,1-Y,1-*Z*); 3.699 (3), 3.415 (2) and 1.421 Å (C3–C8 to C3–C8; symmetry code: 2-*X*,1-Y,1-*Z*); 3.964 (3), 3.615 (2) and 1.626 Å (C10–C15 to C10–C15; symmetry code: 1-*X*,2-Y, 2-*Z*).

### Experimental

In a 10 ml Emrys reaction vial, 4-(2,4-dichlorobenzylidene)-2-phenyloxazol -5(4*H*)-one (0.32 g, 1 mmol), 4,5-dichlorobenzene-1,2-diamine (0.18 g, 1 mmol), TFA (0.23 g, 2 mmol) and ethylene glycol (1.5 ml) were mixed and then capped (The automatic mode stirring helped the mixing and uniform heating of the reactants). The mixture was heated for 16 min at 393 K under microwave irradiation. Upon completion, monitored by TLC, the reaction mixture was cooled to room temperature. The solid product was poured into water and neutralized with 10% NaOH, and then collected by filtration, subsequently washed with ethanol and ethylether in sequence to give a pure yellow solid. A single-crystal suitable for X-ray diffraction was obtained from the evaporation of a solution of the title compound in ethanol.

### Refinement

All H atoms were placed in calculated positions, with N—H = 0.86 Å, and C—H=0.93 Å or 0.97 Å and included in the final cycles of refinement using a riding model, with Uĩso~(H) = 1.2U~eq~(parent atom).

### Crystal data

**Table:**

C_15_H_8_Cl_4_N_2_O	*Z* = 2
*M**_r_* = 374.03	*F*(000) = 376
Triclinic, *P*1	*D*_x_ = 1.663 Mg m^−^^3^
Hall symbol: -P 1	Mo *K*α radiation, λ = 0.71073 Å
*a* = 7.7150 (7) Å	Cell parameters from 742 reflections
*b* = 8.2058 (8) Å	θ = 2.9–26.1°
*c* = 11.9722 (12) Å	µ = 0.79 mm^−^^1^
α = 83.771 (1)°	*T* = 298 K
β = 84.362 (1)°	Prism, colorless
γ = 84.298 (2)°	0.16 × 0.09 × 0.05 mm
*V* = 746.79 (12) Å^3^	

### Data collection

**Table:**

Bruker SMART CCD area-detector diffractometer	2590 independent reflections
Radiation source: fine-focus sealed tube	1364 reflections with *I* > 2σ(*I*)
Graphite monochromator	*R*_int_ = 0.036
φ and ω scans	θ_max_ = 25.0°, θ_min_ = 2.5°
Absorption correction: multi-scan (*SADABS*; Sheldrick, 1996)	*h* = −9→5
*T*_min_ = 0.884, *T*_max_ = 0.961	*k* = −9→9
3811 measured reflections	*l* = −13→14

### Refinement

**Table:**

Refinement on *F*^2^	Primary atom site location: structure-invariant direct methods
Least-squares matrix: full	Secondary atom site location: difference Fourier map
*R*[*F*^2^ > 2σ(*F*^2^)] = 0.055	Hydrogen site location: inferred from neighbouring sites
*wR*(*F*^2^) = 0.103	H-atom parameters constrained
*S* = 1.01	*w* = 1/[σ^2^(*F*_o_^2^) + (0.0247*P*)^2^] where *P* = (*F*_o_^2^ + 2*F*_c_^2^)/3
2590 reflections	(Δ/σ)_max_ < 0.001
199 parameters	Δρ_max_ = 0.27 e Å^−^^3^
0 restraints	Δρ_min_ = −0.26 e Å^−^^3^

### Special details

**Table:**

Experimental. The data was obtained at Xuzhou Medical College collected by Jinpeng Zhang.
Geometry. All e.s.d.'s (except the e.s.d. in the dihedral angle between two l.s. planes) are estimated using the full covariance matrix. The cell e.s.d.'s are taken into account individually in the estimation of e.s.d.'s in distances, angles and torsion angles; correlations between e.s.d.'s in cell parameters are only used when they are defined by crystal symmetry. An approximate (isotropic) treatment of cell e.s.d.'s is used for estimating e.s.d.'s involving l.s. planes.
Refinement. Refinement of *F*^2^ against ALL reflections. The weighted *R*-factor *wR* and goodness of fit *S* are based on *F*^2^, conventional *R*-factors *R* are based on *F*, with *F* set to zero for negative *F*^2^. The threshold expression of *F*^2^ > σ(*F*^2^) is used only for calculating *R*-factors(gt) *etc*. and is not relevant to the choice of reflections for refinement. *R*-factors based on *F*^2^ are statistically about twice as large as those based on *F*, and *R*- factors based on ALL data will be even larger.

### Fractional atomic coordinates and isotropic or equivalent isotropic displacement parameters (Å^2^)

**Table:**

	*x*	*y*	*z*	*U*_iso_*/*U*_eq_	
Cl1	0.77372 (19)	0.40780 (15)	0.26074 (9)	0.0645 (4)	
Cl2	0.62590 (18)	0.18395 (14)	0.47312 (10)	0.0575 (4)	
Cl3	0.4357 (2)	1.04383 (15)	0.75266 (10)	0.0745 (5)	
Cl4	0.0594 (2)	0.7248 (2)	1.09562 (11)	0.0880 (5)	
N1	0.8827 (5)	0.8183 (4)	0.5316 (3)	0.0447 (10)	
H1	0.9289	0.8757	0.4739	0.054*	
N2	0.7257 (5)	0.6358 (4)	0.7161 (3)	0.0431 (10)	
O1	0.9408 (4)	1.0040 (4)	0.6487 (2)	0.0540 (9)	
C1	0.8754 (6)	0.8778 (5)	0.6340 (3)	0.0406 (12)	
C2	0.7819 (6)	0.7756 (5)	0.7279 (3)	0.0418 (12)	
C3	0.7456 (6)	0.5778 (5)	0.6094 (3)	0.0374 (11)	
C4	0.8206 (6)	0.6717 (5)	0.5149 (3)	0.0356 (11)	
C5	0.8302 (6)	0.6185 (5)	0.4072 (3)	0.0416 (12)	
H5	0.8779	0.6820	0.3448	0.050*	
C6	0.7680 (6)	0.4710 (5)	0.3953 (3)	0.0420 (12)	
C7	0.6994 (6)	0.3725 (5)	0.4901 (4)	0.0399 (11)	
C8	0.6891 (6)	0.4266 (5)	0.5954 (3)	0.0419 (12)	
H8	0.6440	0.3613	0.6578	0.050*	
C9	0.7583 (7)	0.8441 (6)	0.8411 (3)	0.0584 (14)	
H9A	0.8507	0.7934	0.8862	0.070*	
H9B	0.7706	0.9614	0.8296	0.070*	
C10	0.5845 (7)	0.8167 (5)	0.9056 (3)	0.0426 (12)	
C11	0.4300 (8)	0.9005 (5)	0.8721 (3)	0.0507 (14)	
C12	0.2672 (7)	0.8752 (5)	0.9292 (4)	0.0544 (14)	
H12	0.1657	0.9328	0.9045	0.065*	
C13	0.2608 (7)	0.7620 (6)	1.0233 (4)	0.0562 (14)	
C14	0.4100 (7)	0.6797 (6)	1.0607 (4)	0.0549 (14)	
H14	0.4037	0.6059	1.1253	0.066*	
C15	0.5711 (7)	0.7057 (5)	1.0027 (3)	0.0517 (14)	
H15	0.6717	0.6485	1.0288	0.062*	

### Atomic displacement parameters (Å^2^)

**Table:**

	*U*^11^	*U*^22^	*U*^33^	*U*^12^	*U*^13^	*U*^23^
Cl1	0.0842 (11)	0.0672 (9)	0.0442 (7)	−0.0149 (8)	0.0021 (7)	−0.0150 (6)
Cl2	0.0672 (10)	0.0447 (7)	0.0626 (8)	−0.0132 (7)	−0.0054 (7)	−0.0079 (6)
Cl3	0.1159 (14)	0.0603 (8)	0.0459 (7)	−0.0194 (9)	−0.0035 (8)	0.0086 (6)
Cl4	0.0741 (12)	0.1161 (13)	0.0705 (9)	−0.0285 (10)	0.0238 (8)	−0.0039 (8)
N1	0.059 (3)	0.042 (2)	0.031 (2)	−0.014 (2)	0.0130 (18)	0.0019 (17)
N2	0.050 (3)	0.050 (2)	0.030 (2)	−0.012 (2)	0.0019 (19)	−0.0031 (18)
O1	0.070 (3)	0.0498 (19)	0.0455 (18)	−0.0295 (19)	0.0063 (17)	−0.0050 (15)
C1	0.041 (3)	0.045 (3)	0.035 (3)	−0.009 (2)	0.001 (2)	0.001 (2)
C2	0.045 (3)	0.049 (3)	0.031 (2)	−0.012 (3)	0.003 (2)	−0.004 (2)
C3	0.039 (3)	0.037 (2)	0.035 (2)	−0.005 (2)	0.005 (2)	0.000 (2)
C4	0.034 (3)	0.032 (2)	0.038 (2)	−0.004 (2)	0.002 (2)	0.003 (2)
C5	0.049 (3)	0.038 (3)	0.035 (2)	−0.008 (2)	0.003 (2)	0.004 (2)
C6	0.039 (3)	0.049 (3)	0.038 (2)	−0.003 (2)	−0.003 (2)	−0.004 (2)
C7	0.041 (3)	0.032 (2)	0.046 (3)	−0.004 (2)	0.000 (2)	−0.005 (2)
C8	0.046 (3)	0.039 (3)	0.037 (3)	−0.009 (2)	0.004 (2)	0.007 (2)
C9	0.072 (4)	0.069 (3)	0.041 (3)	−0.036 (3)	0.002 (3)	−0.013 (2)
C10	0.057 (4)	0.043 (3)	0.032 (3)	−0.022 (3)	0.006 (3)	−0.009 (2)
C11	0.087 (5)	0.037 (3)	0.030 (3)	−0.014 (3)	−0.002 (3)	−0.004 (2)
C12	0.061 (4)	0.055 (3)	0.048 (3)	−0.011 (3)	0.003 (3)	−0.013 (3)
C13	0.068 (4)	0.056 (3)	0.046 (3)	−0.018 (3)	0.014 (3)	−0.017 (3)
C14	0.069 (4)	0.060 (3)	0.037 (3)	−0.024 (3)	0.007 (3)	−0.003 (2)
C15	0.067 (4)	0.053 (3)	0.036 (3)	−0.012 (3)	−0.001 (3)	−0.004 (2)

### Geometric parameters (Å, º)

**Table:**

Cl1—C6	1.740 (4)	C5—H5	0.9300
Cl2—C7	1.738 (4)	C6—C7	1.411 (5)
Cl3—C11	1.750 (4)	C7—C8	1.374 (5)
Cl4—C13	1.740 (5)	C8—H8	0.9300
N1—C1	1.362 (5)	C9—C10	1.505 (6)
N1—C4	1.380 (5)	C9—H9A	0.9700
N1—H1	0.8600	C9—H9B	0.9700
N2—C2	1.292 (5)	C10—C11	1.388 (6)
N2—C3	1.401 (5)	C10—C15	1.399 (5)
O1—C1	1.233 (5)	C11—C12	1.393 (6)
C1—C2	1.498 (5)	C12—C13	1.380 (6)
C2—C9	1.511 (5)	C12—H12	0.9300
C3—C8	1.387 (5)	C13—C14	1.365 (6)
C3—C4	1.407 (5)	C14—C15	1.386 (6)
C4—C5	1.398 (5)	C14—H14	0.9300
C5—C6	1.371 (5)	C15—H15	0.9300
			
C1—N1—C4	124.0 (3)	C7—C8—H8	119.8
C1—N1—H1	118.0	C3—C8—H8	119.8
C4—N1—H1	118.0	C10—C9—C2	113.7 (4)
C2—N2—C3	119.1 (3)	C10—C9—H9A	108.8
O1—C1—N1	123.2 (4)	C2—C9—H9A	108.8
O1—C1—C2	122.7 (4)	C10—C9—H9B	108.8
N1—C1—C2	114.1 (4)	C2—C9—H9B	108.8
N2—C2—C1	123.6 (4)	H9A—C9—H9B	107.7
N2—C2—C9	120.6 (4)	C11—C10—C15	116.8 (4)
C1—C2—C9	115.8 (4)	C11—C10—C9	121.7 (4)
C8—C3—N2	120.0 (3)	C15—C10—C9	121.5 (5)
C8—C3—C4	119.0 (4)	C10—C11—C12	122.8 (4)
N2—C3—C4	121.0 (4)	C10—C11—Cl3	119.6 (4)
N1—C4—C5	121.1 (3)	C12—C11—Cl3	117.5 (4)
N1—C4—C3	118.0 (3)	C13—C12—C11	118.1 (5)
C5—C4—C3	120.9 (4)	C13—C12—H12	121.0
C6—C5—C4	118.8 (4)	C11—C12—H12	121.0
C6—C5—H5	120.6	C14—C13—C12	120.9 (5)
C4—C5—H5	120.6	C14—C13—Cl4	119.6 (4)
C5—C6—C7	120.7 (4)	C12—C13—Cl4	119.4 (5)
C5—C6—Cl1	118.8 (3)	C13—C14—C15	120.3 (5)
C7—C6—Cl1	120.4 (3)	C13—C14—H14	119.8
C8—C7—C6	119.9 (4)	C15—C14—H14	119.8
C8—C7—Cl2	120.2 (3)	C14—C15—C10	121.0 (5)
C6—C7—Cl2	119.9 (3)	C14—C15—H15	119.5
C7—C8—C3	120.5 (4)	C10—C15—H15	119.5
			
C4—N1—C1—O1	175.5 (4)	Cl1—C6—C7—Cl2	1.9 (5)
C4—N1—C1—C2	−4.0 (6)	C6—C7—C8—C3	0.1 (7)
C3—N2—C2—C1	−2.8 (7)	Cl2—C7—C8—C3	−179.2 (3)
C3—N2—C2—C9	178.4 (4)	N2—C3—C8—C7	176.5 (4)
O1—C1—C2—N2	−173.8 (5)	C4—C3—C8—C7	−2.8 (7)
N1—C1—C2—N2	5.7 (7)	N2—C2—C9—C10	−39.4 (6)
O1—C1—C2—C9	5.1 (7)	C1—C2—C9—C10	141.7 (4)
N1—C1—C2—C9	−175.4 (4)	C2—C9—C10—C11	−70.7 (6)
C2—N2—C3—C8	178.8 (4)	C2—C9—C10—C15	109.5 (5)
C2—N2—C3—C4	−2.0 (6)	C15—C10—C11—C12	−1.5 (7)
C1—N1—C4—C5	179.2 (4)	C9—C10—C11—C12	178.8 (4)
C1—N1—C4—C3	−0.2 (7)	C15—C10—C11—Cl3	179.1 (3)
C8—C3—C4—N1	−177.2 (4)	C9—C10—C11—Cl3	−0.7 (6)
N2—C3—C4—N1	3.5 (6)	C10—C11—C12—C13	0.3 (7)
C8—C3—C4—C5	3.4 (7)	Cl3—C11—C12—C13	179.8 (3)
N2—C3—C4—C5	−175.9 (4)	C11—C12—C13—C14	1.2 (7)
N1—C4—C5—C6	179.4 (4)	C11—C12—C13—Cl4	−179.1 (3)
C3—C4—C5—C6	−1.2 (7)	C12—C13—C14—C15	−1.6 (8)
C4—C5—C6—C7	−1.5 (7)	Cl4—C13—C14—C15	178.7 (3)
C4—C5—C6—Cl1	178.0 (3)	C13—C14—C15—C10	0.3 (7)
C5—C6—C7—C8	2.1 (7)	C11—C10—C15—C14	1.1 (6)
Cl1—C6—C7—C8	−177.4 (3)	C9—C10—C15—C14	−179.1 (4)
C5—C6—C7—Cl2	−178.6 (4)		

### Hydrogen-bond geometry (Å, º)

**Table:**

*D*—H···*A*	*D*—H	H···*A*	*D*···*A*	*D*—H···*A*
N1—H1···O1^i^	0.86	1.93	2.789 (4)	173

Symmetry code: (i) −*x*+2, −*y*+2, −*z*+1.
